# Identification of an unknown frameshift variant of *NOG* in a Han Chinese family with proximal symphalangism

**DOI:** 10.1042/BSR20200509

**Published:** 2020-06-15

**Authors:** Zhuang-Zhuang Yuan, Fang Yu, Jie-Yuan Jin, Zi-Jun Jiao, Ju-Yu Tang, Rong Xiang

**Affiliations:** 1 Department of Orthopaedics, Xiangya Hospital of Central South University, Changsha, China; 2 Department of Cell Biology, School of Life Sciences, Central South University, Changsha, China; 3 Human Key Laboratory of Animal Models for Human Diseases, School of Life Sciences, Central South University, Changsha, China

**Keywords:** frameshift variant, NOG, prolonged protein, Proximal symphalangism

## Abstract

Proximal symphalangism (SYM1) is an autosomal dominant disorder manifested by ankylosis of the proximal interphalangeal joints of fingers, carpal and tarsal bone fusion, and conductive hearing loss in some cases. Herein, we clinically diagnosed a Chinese patient with fusions of the bilateral proximal interphalangeal joints in the 2–5 digits without conductive hearing loss. Family history investigation revealed that his mother and grandfather also suffered from SYM1. Whole exome sequencing was performed to detect the genetic lesion of the family. The candidate gene variants were validated by Sanger sequencing. By data filtering, co-segregation analysis and bioinformatics analysis, we highly suspected that an unknown heterozygous frameshift variant (c.635_636insG, p.Q213Pfs*57) in *NOG* was responsible for the SYM1 in the family. This variant was predicted to be deleterious and resulted in a prolonged protein. This finding broadened the spectrum of *NOG* mutations associated with SYM1 and contributed to genetic diagnosis and counseling of families with SYM1.

## Introduction

Proximal symphalangism (SYM1) is a hereditary disorder manifested by ankylosis of the proximal interphalangeal joints, carpal and tarsal bone fusion, and conductive hearing loss in some cases [1]. The typical features of SYM1 are reduced proximal interphalangeal joint space, symphalangism of the 4th and/or 5th finger [2,3]. The estimated prevalence of SYM1 is less than 1/1000000 with autosomal dominant inherited pattern [4,5]. And the first family with ankylosis of the proximal interphalangeal joints was reported and named as symphalangism in 1916 [6].

At present, at least two types of SYM1 have been identified in the clinic. One is proximal symphalangism-1A (SYM1A; OMIM 185800), which was caused by genetic variants in *NOG* (*noggin*), another is proximal symphalangism-1B (SYM1B; OMIM 615298), which resulted from *GDF5* (*growth differentiation factor 5*) mutations [2,7]. However, due to the extensive pleiotropy, several other diseases may be also related to *NOG*, such as tarsal-carpal coalition syndrome, multiple synostoses syndrome, and brachydactyly, etc. [8]. Hence, detection the genetic lesion of the patients with SYM1 may further confirm the clinical diagnosis and help us to understand the development of bone.

In the present study, we enrolled a family with SYM1 from central south region of China. The aim of the present study was to detect the genetic lesion of the affected individuals by employing whole exome sequencing and bioinformatics analysis.

## Materials and methods

### Subjects and ethical approval

**Figure 1:**
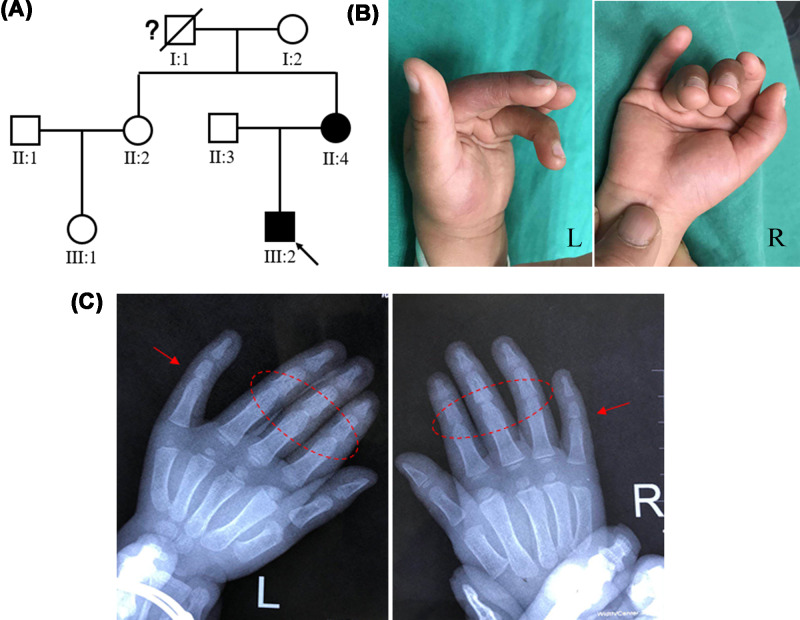
The clinical data of the family with SYM1 (**A**) The pedigree of this family. Black circles/squares are affected, white circles/squares are unaffected. Arrow indicates the proband. The question mark indicates that the illness is uncertain. (**B**) The proband showed the symphalangism of second to fifth fingers. (**C**) Hands X-ray of III-2. The red circles and arrows marked the abnormal regions.

The proband (Figure 1A, III:2) was a 6-year-old boy from a non-consanguineous Chinese family. According to the family history investigation, mother (II:4) and grandfather (I:1) of proband also had the phenotype of limited fingers bilaterally, they may be patients with SYM1. We found the fourth to fifth fingers bilaterally of his mother were limited after preliminary diagnosis. Unfortunately, the proband’s mother refused further diagnosis and treatment and grandfather has already passed away. The photographs showed the second to fifth fingers and toes bilaterally of the proband were limited and cannot make a fist (Figure 1B). The radiographs indicated the reduced proximal interphalangeal joint space and further confirmed the clinical diagnosis (Figure 1C). No other significant phenotypes were found, such as hearing loss.

The Review Board of the Xiangya Hospital of the Central South University approved the present study. Given the proband is too young, written consent forms were signed by his parents as guardians.

### Genetic analysis

Genomic DNA was prepared from peripheral blood of the patients and other all participants using a DNeasy Blood &Tissue Kit (Qiagen, Valencia, CA, U.S.A.). Genomic DNA was extracted from the peripheral blood lymphocytes of all family members by using a DNeasy Blood & Tissue Kit (Qiagen, Valencia, CA, U.S.A.) following the manufacturer’s instruction. The central part of the whole exome sequencing was provided by the Novogene Bioinformatics Institute (Beijing, China). The exomes were captured using Agilent SureSelect Human All Exon V6 kits, and high-throughput sequencing was performed using Illumina HiSeq X-10. The necessary bioinformatics analyses, including reads, mapping, variant detection, filtering, and annotation, were also endowed by Novogene Bioinformatics Institute [9].

The strategies of data filtering refer to our previous study [9]: (a) variants within intergenic, intronic, and UTR regions as well as synonymous mutations were excluded for later analysis; (b) variants with MAF>0.01 in the 1000 Genomes project, dbSNP132 were excluded; (c) variants with MAF>0.01 in genome aggregation database (gnomAD) (http://gnomad.broadinstitute.org/) were further precluded; (d) SIFT, Polyphen-2 and MutationTaster were utilized to predict the possible impacts of variants. (e) Co-segregation analysis was conducted in the family.

## Result

**Figure 2:**
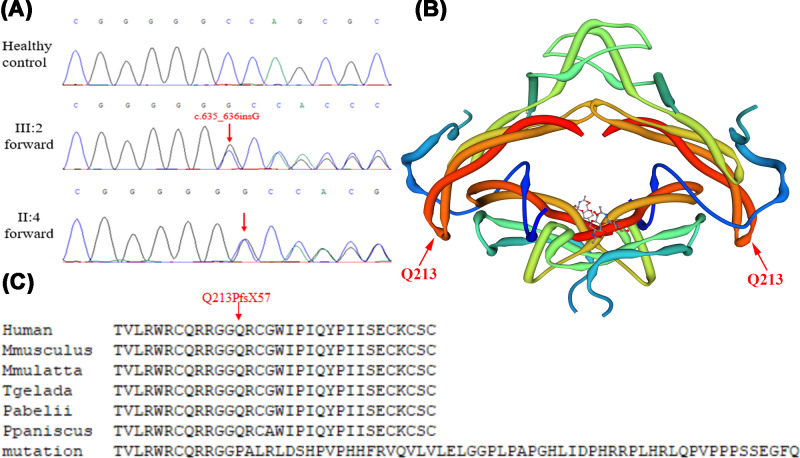
The genetic analysis of the variant (**A**) Sanger DNA sequencing chromatogram demonstrates the heterozygosity for a *NOG* variant (c.635_636insG, p.Q213Pfs*57). (**B**) Rope diagram of noggin–BMP7 complex (SMTL ID: 1m4u.1), the upper and lower parts are noggin dimer and BMP7 dimer, respectively. The arrows and words indicate the Q213 site, the red amino acids rope after Q213 was affected in the patient. (**C**) Alignment of the amino acid sequences of noggin. The affected amino acids locate in the highly conserved amino acid region in different species (from Ensembl). The arrow and words show the Q213 site.

The WES raw data had a mean depth of 125.66 on target, target region coverage of 98.05%, target region coverage (at least 10×) of 97.27%, indicating the high sequencing quality. After data filtering, only 16 variants were included in Table 1. We then further performed bioinformatic analysis including Inheritance pattern and OMIM clinical phenotypes analysis (https://www.omim.org/), ToppGene gene function analysis (https://toppgene.cchmc.org/) and The American College of Medical Genetics and Genomics (ACMG) classification, we highly suspected the unknown variant (NM_005450, c.635_636insG, p.Q213Pfs*57) of *NOG*, belonging to PM1, PM2, PM4, PP1, PP3, and PP4 (likely pathogenic) in ACMG guidelines [10], was the genetic lesion of the family (Figure 2A). The result of co-segregation analysis showed the same unknown variant exist in mother of proband but not in his father. The unknown variant, which led to alteration of amino acid residues after position 212 and a prolonged protein (Figure 2B), was predicted as “Disease Causing” (0.99) by MutationTaster (http://www.mutationtaster.org/) and not found on the 1000 Genome Browser, the gnomAD Browser and the Exome Variant Server, and was not presented in 200 control cohorts. Multiple alignment of noggin orthologs in other animal species showed that amino acid sequence after position 212 was highly conserved (Figure 2C).

**Table 1:** The gene list after data filtering in the family with SYM1

Chr	Pos	RB	AB	Gene	Mutation	OMIM	Allele frequency	Topp gene	ACMG	
1	220275877	C	T	IARS2	NM_018060; c.C790T: p.H264Y	AR: growth hormone deficiency	Unknown variant	Isoleucyl-tRNA aminoacylation	PM2, BP6	Uncertain significance
2	196681652	A	G	DNAH7	NM_018897; c.T9461C:p.V3154A	-	Unknown variant	Inner dynein arm assembly	PM2, PP1, PP3	Uncertain significance
2	233388655	G	A	PRSS56	NM_001195129; c.G1186A: p.E396K	AR: microphthalmia	Unknown variant	Serine-type endopeptidase activity	PM2, BP6	Uncertain significance
5	118485627	C	T	DMXL1	NM_001290321; c.C4105T:p.R1369C	–	Unknown variant	Vacuolar acidification	PM2, PP1, PP3	Uncertain significance
8	99116733	A	G	HRSP12	NM_005836; c.T335C: p.V112A	–	Unknown variant	–	PM2, PP1, PP3	Uncertain significance
9	119461126	G	A	TRIM32	NM_001099679; c.G1105A: p.G369R	AR: Bardet–Biedl syndrome	Unknown variant	Tat protein binding	PM2, BP6	Uncertain significance
12	992601	T	G	WNK1	NM_014823; c.T2789G: p.F930C	AR: neuropathy; AD: pseudohypoaldosteronism	Unknown variant		PM1, PM2	Uncertain significance
12	49522372	A	C	TUBA1B	NM_006082; c.T725G: p.L242R	-	Unknown variant	Chloride channel inhibitor activity	PM2, PP1, PP3	Uncertain significance
17	54672219	G	GG	NOG	NM_005450: c.635_636insG: p. Q213PfsX57	AD: symphalangism	Unknown variant	Fibroblast growth factor receptor signaling pathway	PM1, PM2, PM4, PP1, PP3, PP4	Likely pathogenic
17	71420107	G	A	SDK2	NM_001144952; c.C1708T: p.R570W	–	Unknown variant	Camera-type eye photoreceptor cell differentiation	PM2, PP1, PP3	Uncertain significance
18	13071096	G	A	CEP192	NM_032142; c.G5233A: p.E1745K	–	Unknown variant	Phosphatase binding	PM2, PP1, PP3	Uncertain significance
19	39321974	T	G	ECH1	NM_001398; c.A235C: p.N79H	–	Unknown variant	Δ3,5-Δ2,4-Dienoyl-CoA isomerase activity	PM2, PP1, PP3	Uncertain significance
19	56128115	T	C	ZNF865	NM_001195605; c.T3131C: p.L1044P	–	Unknown variant	–	PM2, PP1, PP3	Uncertain significance
20	25457050	C	CA	NINL	NM_025176; c.2876_2877insT: p.E959Dfs15	–	Unknown variant	Calcium ion binding	PM2, PP1, PP3	Uncertain significance
20	30785333	G	A	PLAGL2	NM_002657; c.C413T: p.T138M	–	Unknown variant	Chylomicron assembly	PM2, PP1, PP3	Uncertain significance
X	131188838	G	T	STK26	NM_001042452; c.G222T:p.L74F	–	Unknown variant	Microvillus assembly	PM2, PP1, PP3	Uncertain significance

CHR, chromosome; POS, position; RB, reference sequence base; AB, alternative base identified; AR, autosomal recessive; AD, autosomal dominant; BP, benign supporting; PP, pathogenicity supporting; PM, pathogenicity moderate; PVS, pathogenicity very strong. The data of allele frequency were obtained from 1000G, ESP, and ExAC databases.

## Discussion

In the present study, we enrolled a family with SYM1 from China. By employing whole exome sequencing, we identified an unknown frameshift variant (c.635_636insG, p.Q213Pfs*57) in the affected members. The variant resulted in the extension of noggin protein which may affect the function of the protein. Bioinformatics analysis further predicted this variant as disease-causing variant. Our study is consistent with previous studies which indicated that variants in *NOG* gene may lead to SYM1 and other bone diseases [11].

The human *NOG* gene encoding noggin protein is located on chromosome 17q22, and it consists of one exon, spanning approximately 1.9 kilobases (kb). Noggin, the first identified BMP antagonist, is posttranslationally modified and secreted as a disulfide-bonded homodimer. BMPs play essential roles in skeletogenesis including recruiting mesenchymal cells, promoting mesenchymal cell proliferation and differentiation into chondroblasts and osteoblasts, and inducing apoptosis to form joints [12–14]. Noggin can bind to BMPs and inhibit the interactions of BMPs and BMP-specific recptors, and therefore negatively regulates BMP-induces osteogenesis [15,16]. In the present study, the unknown variant was not located at the interface between the two molecules in noggin–BMP7 complex (SWISS-MODEL Template Library, ID: 1m4u.1), and no templates of sufficient quality to build a homology model were found for the changed sequence (Figure 2B). Whereas, according to the complex model and the prolonged sequence, we suspected the variant presumably affected the binding of noggin homodimer and further disrupt the structure of noggin–BMP7 complex, which actived the BMP signal pathway and lead to bone diseases. Further research is needed to confirm this hypothesis.

On the basis of reported papers, multiple bone diseases are associated with *NOG* mutations [17]. For example, at present, over 50 mutations of *NOG* involved in wide variety of bone development anomalies, including tarsal/carpal coalition syndrome, brachydactyly, multiple synostoses syndrome, stapes ankylosis with broad thumbs and toes, have been reported [5,18]. Even the same variants of *NOG* can lead to different phenotypes between different families or different affected members of the same family, see Table 2 [19,20]. Meanwhile, the variant was the sixth unknown variant reported in Chinese population, which indicated there were still a lot of unknown variants to be discovered in Chinese population. Here, we summarized the reported *NOG* mutations in Table 2, which may make us to understand the function of noggin better.

**Table 2:** The summary of reported mutations in NOG

No.	Mutation		Phenotypes			PMID
1	c. 58delC	p. Leu20fs	SYNS1	Hearing loss	–	11846737
2	c. 103C>G	p. Pro35Ala	BDB	–	–	17668388
3	c. 103C>T	p. Pro35Ser	TCC	Hearing loss	Hyperopia	18440889
4	c. 103C>T	p. Pro35Ser	SYM1	Hearing loss	–	11857750
5	c. 103C>T	p. Pro35Ser	BDB	–	–	17668388
6	c. 104C>G	p. Pro35Arg	SYM1	–	–	10080184
7	c. 104C>G	p. Pro35Arg	TCC	–	–	11545688
8	c. 106G>C	p. Ala36Pro	BDB	–	–	17668388
9	c. 110C>G	p. Pro37Arg	TCC	Hearing loss	–	15264296
10	c. 124C>G; c. 149C>G	p. Pro42Ala; p. Pro50Arg	TCC	Hearing loss	–	15736221
11	c. 124C>T	p. Pro42Ser	SYM1	–	–	31370824
12	c. 125C>G	p. Pro42Arg	SYNS1	–	–	18204269
13	c. 124C>A	p. Pro42Thr	SYNS1	–	–	23732071
14	c. 125C>T	p. Pro42Leu	SYNS1	Hearing loss	–	25241334
15	c.130_131insGG	p. Val44fs	TCS	Hearing loss	Hyperopia	15699718
16	c. 137T>C	p. Leu46Pro	SYM1	–	–	22855651
17	c. 142G>A	p. Glu48Lys	BDB	–	–	17668388
18	c. 142G>A	p. Glu48Lys	POF and SYM1	Hearing loss	–	15066478
19	c. 163G>T	p. Asp55Tyr	SYM1	–	–	31105738
20	c. 252_253insG	p. Glu85fs	SABTT	Hearing loss	Hyperopia	12089654
21	c. 261_262insG	p. Pro88fs	SYNS1	Hearing loss	Hyperopia	25241334
22	c. 271G>T	p. Gly91Cys	FOP	–	–	11503156
23	c. 274G>C	p. Gly92Arg	FOP	–	–	11503156
24	c. 275G>A	p. Gly92Glu	FOP	–	–	11503156
25	c. 283G>A	p. Ala95Thr	FOP	–	–	16080294
26	c. 304delG	p. Ala102fs	SYM1	Hearing loss	Hyperopia	21358557
27	c. 328C>T	p. Gln110X	SABTT	Hearing loss	Hyperopia	12089654
28	c. 386T>A	p. Leu129X	SYM1	Hearing loss	–	11846737
29	c. 391C>T	p.Gln131X	SABTT	Hearing loss	Hyperopia	21358557
30	c. 397A>T	p. Lys133X	SABTT	Hearing loss	Hyperopia	27508084
31	c. 406C>T	p. Arg136Cys	SYM1	Hearing loss	–	24735539
32	c. 450G>C	p. Trp150Cys	SYM1	Hearing loss	–	25888563
33	c. 452C>A	p. Ser151X	SYNS1	Hearing loss	–	25241334
34	c. 463T>A	p. Cys155Ser	SYM1	Hearing loss	–	22288654
35	c. 499C>G	p. Arg167Gly	BDB	–	–	17668388
36	c. 499C>T	p. Arg167Cys	SYM1	–	–	24326127
37	c. 551G>A	p. Cys184Tyr	SYM1	–	–	11846737
38	c. 551G>T	p. Cys184Phe	SYM1	Hearing loss	Hyperopia	22288654
39	c. 559C>T	p. Pro187Ser	BDB	–	–	17668388
40	c. 559C>G	p. Pro187Ala	SYM1	Hearing loss	–	25391606
41	c. 561delC	p. Pro187fs	TCS	Hearing loss	Hyperopia	15699718
42	c. 565G>T	p. Gly189Cys	SYM1	–	–	10080184
43	c. 568A>G	p. Met190Val	SYNS1	Hearing loss	–	18204269
44	c. 608T>C	p. Leu203Pro	TCS	Hearing loss	Hyperopia	15699718
45	c. 611G>T	p. Arg204Leu	TCC	–	–	11545688
46	c. 611G>G	p. Arg204Gln	TCC	–	–	29159868
47	c. 614G>A	p. Trp205X	SYNS1	–	–	16532400
48	c. 615G>C	p. Trp205Cys	Facioaudiosymphalangism syndrome	Hearing loss	Hyperopia	15770128
49	c. 615G>C	p. Trp205Cys	SABTT	Hearing loss	Hyperopia	19471170
50	c. c.635_636insG	p.Q213PfsX57	SYM1	–	–	Present study
51	c. 645C>A	p. Cys215X	SABTT	Hearing loss	Hyperopia	22288654
52	c. 649T>G	p. Trp217Gly	SYNS1	–	–	10080184
53	c. 659T>A	p. Ile220Asn	SYM1	–	–	10080184
54	c. 659_660delinsAT	p. Ile220Asn	SYM1	–	–	10080184
55	c. 664T>G	p. Tyr222Asp	SYM1	–	–	10080184
56	c. 665A>G	p. Tyr222Cys	SYM1	–	–	10080184
57	c. 665A>G	p. Tyr222Cys	TCC	–	–	11545688
58	c. 668C>T	p. Pro223Leu	SYM1	–	–	10080184
59	c. 682T>G	p. Cys228Gly	SABTT	Hearing loss	Hyperopia	26211601
60	c. 682T>A	p. Cys228Ala	SYNS1	Hearing loss	Hyperopia	25391606
61	c. 689G>A	p. Cys230Tyr	SYNS1	–	Hyperopia	26994744
62	c. 690C>G	p. Cys230Trp	SYM1	Hearing loss	–	31694554
63	c. 696C>G	p. Cys232Trp	SYNS1	Hearing loss	Hyperopia	20503332

SYNS1, multiple synostosis syndrome; BDB, brachydactyly type B; TCC, Trasal–Carpal coalition syndrome; SYM1, proximal symphalangism; TCS, Teunissen–Cremers syndrome; POF, premature ovarian failure; SABTT, stapes ankylosis with broad thumbs and toes; FOP, fibrodysplasia ossificans progressiva.

In additional to major bone diseases, patients with *NOG* mutations are often accompanied by other phenotypes, such as conductive hearing loss and hyperopia. In Table 2, we can find that these phenotypes are not always present in the same mutations or in different mutations at the same sites. Besides, in some papers, hearing loss do not exist in all affected members of same families [5,18]. These results seem to indicate that conductive hearing loss and hyperopia may appear randomly in patients with *NOG* mutations; whereas, in contrast with most NOG mutations that have been reported in kindreds with SYM1 and SYNS1, the mutations observed in families with stapes ankylosis without SYM1 are predicted to disrupt the cysteine-rich C-terminal domain [21,22]. In short, the relationship between *NOG* and these phenotypes is still unclear, further research is needed to understand that. Some patients with *NOG* mutations can also have nasal bone, elbow, shoulder, and spine anomalies except for hands and feet [11,14], suggested noggin protein plays an essential and extensive role in bone development.

In summary, we investigated a Chinese family with SYM1 and an unknown frameshift variant (c.635_636insG, p.Q213Pfs*57) was detected by whole exome sequencing. According to ACMG standards and guidelines, this variant was categorized as likely pathogenic (PM1, PM2, PM4, PP1, PP3 and PP4) and identified as the genetic lesion of the family. Our study expanded the spectrum of *NOG* mutations and contributed to genetic counseling and diagnosis of patients with SYM1.

## Abbreviations

ACMG: The American College of Medical Genetics and Genomics
GDF5: growth differentiation factor 5
gnomAD: genome aggregation database
NOG: noggin
OMIM: Online Mendelian Inheritance in Man
SYM1: proximal symphalangism
SYM1A: proximal symphalangism-1A
SYM1B: proximal symphalangism-1B
SYNS1: multiple synostosis syndrome
